# Environmental, Individual and Personal Goal Influences on Older Adults’ Walking in the Helsinki Metropolitan Area

**DOI:** 10.3390/ijerph16010058

**Published:** 2018-12-26

**Authors:** Tiina E. Laatikainen, Mohammad Haybatollahi, Marketta Kyttä

**Affiliations:** 1 Department of Built Environment, Aalto University, P.O. BOX. 14400, 00076 Aalto, Finland; marketta.kytta@aalto.fi; 2 Department of Public Health, University of Helsinki, 00014 Helsinki, Finland; mohammad.haybatollahi@helsinki.fi

**Keywords:** walking, active travel, ageing, physical environment, personal projects, activity space, Public Participatory GIS (PPGIS)

## Abstract

Physical activity is a fundamental factor in healthy ageing, and the built environment has been linked to individual health outcomes. Understanding the linkages between older adult’s walking and the built environment are key to designing supportive environments for active ageing. However, the variety of different spatial scales of human mobility has been largely overlooked in the environmental health research. This study used an online participatory mapping method and a novel modelling of individual activity spaces to study the associations between both the environmental and the individual features and older adults’ walking in the environments where older adult’s actually move around. Study participants (*n* = 844) aged 55+ who live in Helsinki Metropolitan Area, Finland reported their everyday errand points on a map and indicated which transport mode they used and how frequently they accessed the places. Respondents walking trips were drawn from the data and the direct and indirect effects of the personal, psychological as well as environmental features on older adults walking were examined. Respondents marked on average, six everyday errand points and walked for transport an average of 20 km per month. Residential density and the density of walkways, public transit stops, intersections and recreational sports places were significantly and positively associated with older adult’s walking for transport. Transit stop density was found having the largest direct effect to older adults walking. Built environment had an independent effect on older adults walking regardless of individual demographic or psychological features. Education and personal goals related to physical activities had a direct positive, and income a direct negative, effect on walking. Gender and perceived health had an indirect effect on walking, which was realized through individuals’ physical activity goals.

## 1. Introduction

Extensive evidence exists that physical activity (PA) has notable health benefits for older adults [1,2,3,4]. In addition, maintaining mobility—one’s ability to move around and take care of everyday activities—is a fundamental factor in healthy aging [5,6]. Research has also shown that active travel (AT), namely walking and cycling, has health benefits across population even after adjustment for other forms of PA [7]. In their recent review, Cerin and colleagues [8] found strong links between the neighborhood physical environment and older adults’ AT. Thus, it is of prime importance to ensure that older adults can sustain mobility in their everyday environments.

According to the ecological models of health behavior [9,10] multiple levels of factors influence human health behavior, often including intrapersonal, interpersonal, organizational, community, physical environmental, and policy. These factors work together and influences interact across different levels, meaning that individuals with high motivation for sports might react differently to new bike lanes implemented to their neighborhood than those who are not very interested in sports living in the same area [11]. According to Sallis and Owen [11] studies with multilevel approach should explain health behaviors better than studies that focus only on single level. Despite this notion, previous research has concentrated mainly on identifying either individual or physical environmental factors related to PA in general or to some specific domain of PA in particular.

Research focusing on the associations between individual factors and PA have found a host of individual characteristics associated with older adults’ PA [12,13,14]. Aside from the individual demographic factors, a few studies have examined the associations between PA and intrapersonal factors, such as motivation and self-efficacy [14,15,16,17]. Studies examining associations between individual goal setting and PA conclude that having specific health- and PA-related goals is an important component to increasing exercise and PA in older adults [18,19,20].

Besides studying actual health related goal setting, researchers have studied the interactions between general personal goals, health and PA [21,22,23,24,25,26]. Personal goals, often referred also as personal projects, are defined as intentions that describe motivational features behind people’s actions or states people strive to achieve or avoid in the future [23,24,27,28]. Older adults’ personal goals related to physical activity and cultural functions have been found associated with high exercise activity [23]. According to Little et al. [28] personal projects as analytical units are nested within a larger social ecological framework for personality and developmental science. The social ecological model by Little et al. [28] proposes, rather similarly to the ecological model of health behavior [11], that both personal features as well as environmental features have direct effects as well as indirect influences through personal projects to the outcome measures such as the physical well-being. A few studies have used the social ecological model or the concept of personal goals to explore what features support or hinder PA [22,25]. However, in their systematic review Notthoff and colleagues [14] concluded that studies examining associations between older adults’ intrapersonal factors, such as motivational goals or self-efficacy, and PA are still rather scarce.

Research that focuses simply on the individual influences on PA have been criticized for failing to acknowledge the context where the behavior actually takes place [10,29]. However, the past decade has introduced a growing number of studies that examine the influences of the physical environment on PA [30,31]. Most studies have examined the associations between the neighborhood built and natural environments and health of older adults [32,33,34,35,36]. According to these studies, walkability, connectivity, density, mixed land-use, green and water environments, and closeness to home of everyday destinations are important characteristics of the environments that support healthy aging [6,32,37,38].

However, most of these studies focus simply on the physical environmental factors in the immediate home vicinity and their associations with PA by analyzing the built environment features around individuals’ residences or neighborhoods that have been delineated through administrative units or residential buffers with varying radii and buffering methods [39]. Analyses of people’s everyday mobility behavior and exposure outside their residential neighborhoods have been problematic, leading to flawed interpretations about the health impacts of physical environmental factors [39,40,41]. Also, according to Blacksher and Lovasi [42] there is a lack of research that recognizes that the effect of physical environment on health is subject to human perception.

In this paper we aim to address the gap in research that focuses on the multiple-level influences of health behaviors and examine how multiple levels of factors influence older adults AT. While some individual features, such as gender, motivation and particular PA-related goals [14,19], and on the other hand certain environmental features [8,43] have shown influencing PA among older adults, these features have not been widely studied simultaneously and context-sensitively according to the principals of ecological models [11]. While it is well acknowledged that environmental context can shape or constrain individual determinants of health behavior, there have not been many studies examining the multifaceted influences of the environment and the individual on PA. This is especially true for outside the administrative or residential neighborhoods, perhaps due to considerable methodological challenges [10].

Participatory mapping methods, such as Public Participation Geographic Information System (PPGIS), have offered convenient tools for previous studies investigating the active two-way person-environment relationship [44,45,46,47]. Localization of human experiences and behavioral patterns by advanced public participatory mapping tools attaches them to a specific physical environmental context [48]. Thus, the human behavior and experiences get geographic coordinates, which allows simultaneous GIS-based analysis of human behavior in relation to the physical environment [49]. These kinds of spatial studies on human health behavior has proven effective and the usage of GPS tracking or map-based questionnaires have provided a way to overcome the identified contextual challenges, and improved our understanding about the mechanisms that connect place to health [39,50].

In this study, we examined the individual and physical environmental features that influence older adults’ AT within their everyday environments, including the environment also outside their immediate home vicinity. We examined the AT as older adults’ walking for transport, given the known health benefits and popularity of this particular travel mode among older adults [6,8,51]. We examined the walking of older adults who live in the capital region of Helsinki, Finland and focused on defining how and which of the environmental and individual factors direct their walking.

Previous studies adopting the principles of ecological models have had methodological challenges developing and collecting measures of influences at multiple levels and capturing the complex interactions of individual and physical environmental characteristics [11]. In addition, previous studies on physical environment and health has mainly focused on neighborhood environments, overlooking people’s mobility behavior in non-residential locations [39,40]. To overcome the identified challenges, we used an online participatory mapping method and a novel modelling of individual activity spaces in this study, which enabled us to study simultaneously and context-sensitively the associations between both the individual and the environmental features and older adults’ walking in the environments where they actually move around.

## 2. Materials and Methods

### 2.1. Study Area

The Helsinki Metropolitan Area (HMA) consists of four independent city units, Espoo, Helsinki, Kauniainen and Vantaa. Helsinki is the capital of Finland and forms with its surrounding three cities the HMA region. Finland and its capital region is an interesting and topical case study site due to the rapid population ageing in the country. The share of Finnish people over 65 years old is currently 21.4 percent and is estimated to be 26.4 percent of the population by 2030 and 28.7 percent by 2050 [52]. The ageing phenomena in Helsinki is currently still moderate compared to the whole country. At the beginning of 2018 there were about 25 65-years-olds per 100 working age adults in Helsinki whereas the numbers were 36 per 100 in the whole country. However, the amount 65-years-olds has increased 46 percent in Helsinki during the last decade, whereas the amount general population has increased only 17 percent [53]. GDP per capita (PPS) in HMA was 52.021 € in 2015 [54] and the region is characterized with good public transit connections and accessibility [55]. The pedestrian environment in HMA is generally good. Most of the arterial, collector as well as local roads have separated sidewalks. Sidewalks in the central areas are separated from the bicycle lanes, but in suburban areas, sidewalks are mainly shared between pedestrians and bicyclists. Pedestrian crossings are frequent both in the central urban areas as well as in the suburban areas. Signalled crosswalks are also common, but signals do not show minutes for walking. During winter most of the walkways, including sidewalks, separate pedestrian-only streets, sidewalks shared between pedestrian and bicyclists and common trails are routinely plough and gritted excluding some forest trails and jogging routes.

### 2.2. Participatory Mapping Method

**Figure 1:**
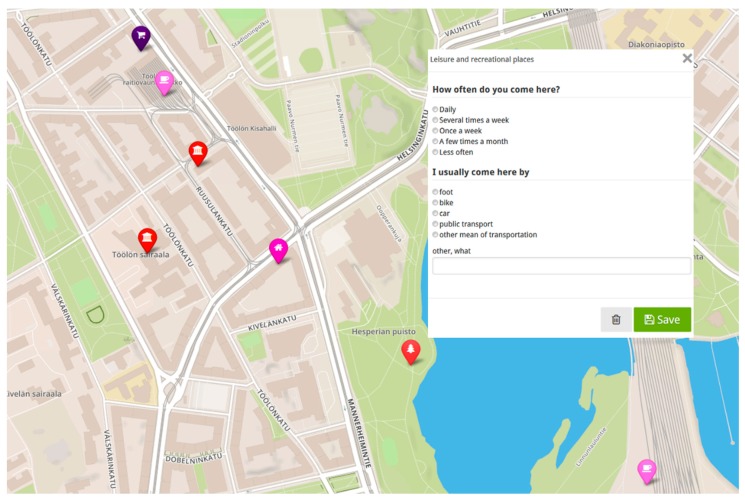
The online interface of the survey.

Data were collected using an online participatory mapping method, PPGIS, which combines internet maps with traditional questionnaires [49]. PPGIS methods were developed for the purposes of both research and participatory planning practice to collect spatial experiential knowledge and to engage non-experts to identify the spatial dimensions of the environment [49]. In our study, respondents used an online interface to mark their (1) everyday errand points (EEPs) on a map (Figure 1). In addition, the respondents indicated which (2) transport mode they used and how (3) frequently they accessed the EEPs. The respondents were asked to mark on a map their (4) home and answer questions related to (5) their personal characteristics, such as their sociodemographic background and perceived health as well as (6) personal psychological features, namely respondents’ personal goals.

With this place-based mapping method, we were able to study older adults’ travel behavior spatially and context-sensitively by asking respondents to pinpoint their everyday behavior on the map. The respondents’ individual characteristics were studied simultaneously with the physical environment by asking them to describe their sociodemographic background and to evaluate the importance of a series of personal goals. Localization of human behavioral patterns by participatory mapping tools attaches them to specific physical environmental context [48]. This way human behavior and experiences receive geographic coordinates, which allows simultaneous GIS-based analysis of human behavior in relation to the physical environment (Figure 1).

### 2.3. Home Range Model Capturing the Walking Behavior

Previous studies interested in the relationship between the built environment and human health, have mainly used static spatial units of analysis to capture the GIS-based physical environmental variables [56]. Administrative boundaries, postal code areas and census tracts are examples of static and simple spatial units of analysis to capture the environmental context. More developed spatial units of analysis are buffers, spherical or network, that are created around individual home locations of study participants [57,58]. All such approaches presume that individual health behavior is bound to static neighborhood boundaries or certain buffered distances around their home. These approaches have been criticized for being too static and not accounting for actual individual differences in mobility exterior to the place of residence since they tend to ignore individual’s true spatio-temporal behavior [39,40,56,59,60]. Recently, researchers have proposed alternative modeling approaches that correspond more to individual activity patterns and are more adaptive in their boundaries and structure [56,60].

**Figure 2:**
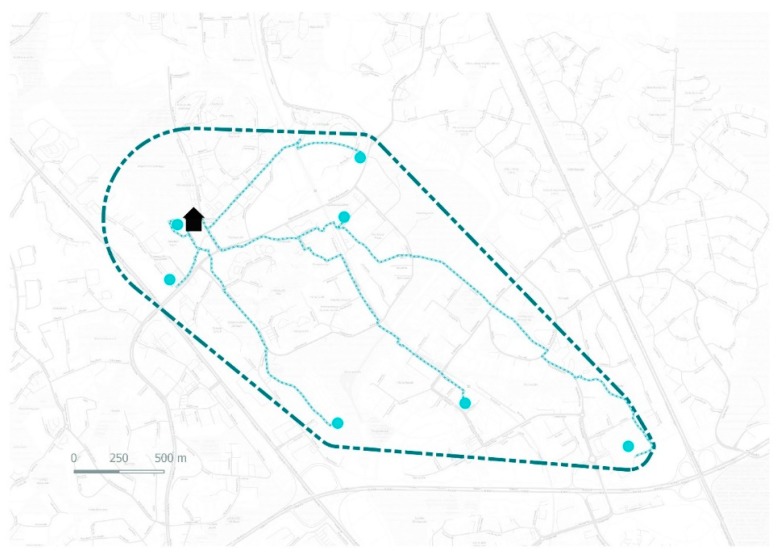
An example of an individual home range and active travel routes of a respondent.

In this study we took a step forward from the static approaches for capturing the contextual effects related to older adults’ active travel. Thus, we applied a dynamic model of home ranges developed by Hasanzadeh and colleagues [56]. The home range model is an individual-specific dynamic boundary method which take into account the individual-specific variations of home ranges, also referred to as activity spaces [56,60]. The model of home ranges is also parametric, meaning that it can be applied for different purposes and studies by specifying its parameters for each individual study purpose (more detailed description of the model parameters in [56]). The model uses customized minimum convex polygons created around individuals’ home and everyday errand points to capture individuals’ neighborhoods instead of plain static administrative boundaries or spherical buffers only around individuals’ homes (Figure 2). In their recent study Laatikainen and colleagues [39] compared different neighborhood and activity space models to capture the physical environment. They found that novel activity space models such as the home range (HR) are in many cases more suitable approaches than static measures like buffers for measuring the physical environment and the activities of individuals and, thus, capturing individual environmental exposure. In their study Laatikainen et al. [39] found that walkability of individual home ranges was positively correlated with perceived wellbeing of older adults but warranted for more studies to investigate how the walkability of the home range is associated with AT. Thus, the home range model was applied in this study to capture the activity spaces of older adults and to study how the physical environment outside plain residential areas affect older adults walking. The home range (HR) was modelled for each respondent and the physical environment features within the HR’s was calculated for each individual (Figure 2).

### 2.4. Conceptual Framework and Hypotheses

**Figure 3:**
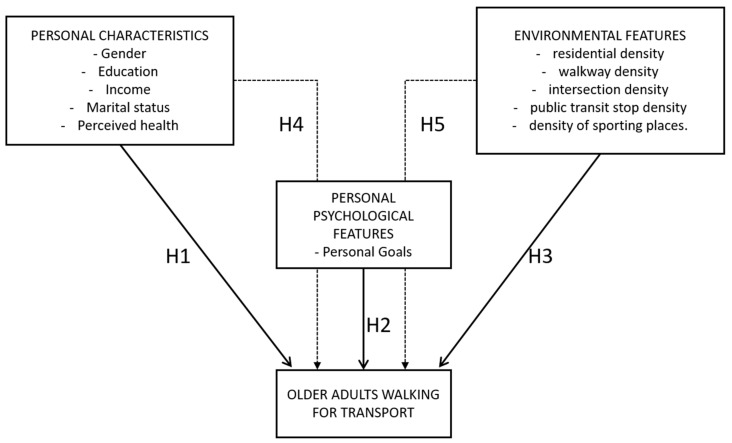
The conceptual framework.

Previous studies have examined the associations between both the individual and the environmental factors and PA, or more specifically the active travel behavior of older adults, concluding that multiple levels of factors affect the PA behaviour [8,11,14]. Following the principles of the ecological models of health behavior [11] and the social ecological model of Little [61] the conceptual framework for this study focuses on the impacts of the personal characteristics, environmental features and personal psychological features on AT of older adults (Figure 3). The framework illustrates the interactions among personal characteristics, different environmental features and personal goals on AT of older adults. The framework proposes that the personal characteristics, the environmental features as well as the psychological features, namely the personal goals, have direct effects, but that there are also indirect influences through personal psychological factors to the AT behavior of older adults. Thus, following the social ecological framework proposed by Little [61] we hypothesize that personal goals serve as mediating conduit through which different personal and environmental features influence the walking behavior of older adults (H4 and H5). In addition, we tested the direct modelling hypothesis by evaluating the direct relationship between the personal characteristics (H1), psychological features (H2) and environmental features (H3) and walking.

### 2.5. Participants

A random sample of 5000 residents of the Helsinki Metropolitan Area (HMA) aged between 55 and 75 received an invitation letter by mail asking them to participate in an online mapping survey. A total of 1,139 full or partial responses were received, and after removing incomplete responses, 844 were taken for further analysis. Participants consisted of 447 women and 331 men with a mean age of 64.3 (SD = 5.52). The data showed general consistency on most sociodemographic variables within the study region (Table 1). The data was collected during early fall 2015. All subjects were informed about the study and its content in a letter inviting them to participate in the online survey. By participating in the survey all participants gave their consent for inclusion. The Research Ethics Committee of Aalto University approved the study protocol.

### 2.6. Measures

*Walking for transport.* A dependent variable of walking was developed using the collected PPGIS data. The measure consisted of the EEP locations marked by participants with corresponding travel mode, frequency of visitation, and a network distance from place of residence to the location. In the survey respondents reported modes of traveling as walking, cycling, driving, or using public transit. Frequency of visitation was reported as daily, several times per week, several times per month, a few times per month, and less than monthly. Distances between home and visited places were calculated as the network distance between the home locations of each respondent and their EEPs (Figure 2). Each distance was weighted based on the frequency of visits per month (daily = 25, several times per week = 12, several times per month = 5, a few times per month = 3, less than monthly = 1). We excluded two days per week for the daily option to be equivalent to the weights of the home range model used in this study, where home is given a monthly visitation value of 30 [56]. Each calculated distance that was traveled by walking was categorized as walking and distances travelled by cycling, public transit or private car were omitted for this study. The final dependent variable was calculated as total monthly walking and is referred to walking hereafter.

*Personal characteristics*. To study the association between personal characteristics and walking, we analyzed respondents’ individual demographic characteristics such as gender, education, income, marital status and perceived health. These particular variables were chosen because they have been linked to older adults PA behavior in previous research [14].

*Personal goals*. We analyzed respondents’ personal goals in order to study the both the direct and indirect associations of older adults’ intrapersonal psychological factors on walking. The personal goals were measured by means of 19 individualized states formulated based on previous extensive literature on older adults’ personal goals [21,22,24,28,61,62,63,64]. In the survey, respondents were asked to rank the importance of the personal goals using a seven-point Likert scale that ranged from 0 (not important) to 6 (very important). The goals are listed hereafter in data analysis and Table 2.

*Physical environment features*. GIS-based variables were used to study the physical environment in relation to respondents’ walking [6,57,65]. In their recent review Cerin and colleagues [8] concluded that older adults’ AT was strongly positively associated with neighborhood walkability. Other previous studies have found PA in general and AT in particular positively associated with residential density, connectivity and density of destinations [6,31,66]. Instead of using the common walkability index [67], we calculated separate physical environment density measures to assess the walkability of the home ranges. This was due to high correlations between the measures of walkability index as well as between the walkability index and the size of the home range. Earlier studies have also highlighted the issues related to modifiable areal unit problem (MAUP) and to multicollinearity issues in the data [68,69]. In addition, using the land-use mix, an integral part of the walkability index, together with rather small spatial units has been found challenging also elsewhere [70]. Thus the following physical environment features were included in the study and calculated as follows:

Walkway density was assessed as the share of walkable streets within the HR. The walkway measure was calculated as the share of walkways in kilometers within the HR. The walkway dataset was drawn from Open Street Map (OSM) which is open geospatial data produced by a community of mappers. The dataset includes all streets that are meant only for walking but also streets that are shared for walking and bicycle as well as sidewalks that are along the side of a road. The data of OSM is fully open and licensed under the Open Data Commons Open Database License (ODbL) by the OpenStreetMap Foundation (OSMF).

Residential density was calculated as residential floor area divided by residential land use within each HR. The residential density measure was drawn from SeutuCD 2014, a regional dataset provided by Helsinki Region Environmental Services Authority HSY.

The connectivity was operationalized with two different measures: as the share of intersections of three or more road segments per individual home range [67] and as the share of public transit stops [8] within HR. The connectivity measures were drawn from the Digiroad 2017 dataset maintained by the Finnish Transport Agency.

The share of *sporting places* within HR was also calculated. The measure includes all sports facilities, recreation areas and hiking trails. The sporting places were drawn from the LIPAS dataset. LIPAS is developed by the Faculty of Sport and Health Sciences, University of Jyväskylä, in collaboration with the Ministry of Education and Culture, the Association of Finnish Local and Regional Authorities, various authorities of regional administration, municipalities, environmental administration, sports federations and other organisations, and the maintainers of sport facilities.

The physical environment variables were extracted and calculated using the ArcMap 10.5 program (Esri, Redlands, CA, USA). We created individual home ranges for each respondent as by the principles of the home range model [56]. Finally, we calculated all of the above listed physical environment variables within each individual home range.

### 2.7. Statistical Analysis

In order to investigate the structure underlying the intrapersonal psychological factors of the respondents, an explanatory factor analysis (EFA) with Promax rotation and Kaiser Normalization was conducted for 12 personal goal variables. Due to low correlations with other goal variables, seven personal goals were left out from the final EFA after careful examination of the correlation matrix. These were goals related to working, self-development, managing with diseases, religion, traveling, handcraft hobbies, and diet. After identifying the components, Anderson-Rubin factor scores were estimated for each participant.

Finally, the associations between walking and sociodemographic background characteristics, the physical environment variables, and goal factors were examined using ordinary least squares (OLS) regression. Direct, indirect, and total effect for mediation analysis were estimated using structural equation modeling without latent variable (or multiple regression) that allows the indirect (mediation) effect to be a product of the reduction of the total and direct effects of predictors on the outcome. The data for total walking, walkway and intersection density were positively skewed, thus we transferred these variables using square root transformation because the data contained zero values. The data were checked if they met the basic and specific assumptions of OLS regression analysis. The residuals were normally distributed, and the variability of the total walking was homoscedastic across the predictors. There were, however, high correlations between the physical environment measures, which indicated the existence of a multicollinearity issue if we used these variables together in a single model. Because only one physical environment measure was used in each model, the observed high correlation between the physical environment variables did not pose a multicollinearity issue to the results. IBM SPSS statistics 25 (IBM Corp, Armonk, NY, USA), Mplus version 7.3 (Program Copyright © 1998-2012 Muthén & Muthén) and statistics version 3.3.0 with R studio (RStudio: Integrated Development for R. RStudio, Inc., Boston, MA, USA) were used to perform the statistical analyses.

## 3. Results

The respondents (*n* = 788) marked, on average, six everyday errand points on the map in the survey and walked on average 20 kilometers per month (SD = 29.9). The descriptive statistics of all the measures used in the further analysis are presented in Table 3.

### 3.1. Older adults’ Personal Goal Factors

An EFA was performed to study the personal goals and their effect on walking alongside the individual and environmental features. After carrying out the EFA analysis, four factors were extracted from the 12 personal goals that explain approximately 62% of the variance (Table 2). Each component was labeled according to their most representative personal goals. As reflected in Table 2, three goals contributed to the first factor. These goals dealt with PA, sports, and health and functional capacity. A high score in this component indicates that the respondent evaluated PA, health, and sports as important personal goals for them. We called this factor “PA and sports.” Three goals related to the health of other people, relatives, and social relationships contributed to the second factor. A high score on this component indicates that the respondent evaluated others’ health and wellbeing and social relationships as very important personal goals for them. This factor was named “caring for others.” Three goals contributed to the third factor. These goals dealt with independent living and the preservation of an independent lifestyle, management of financial issues and/or assets, and maintaining memory capacities. Thus, we labeled the third factor as “manage on one’s own”. Finally, the last component was labeled ”culture and social affairs” because the three goals contributing to this factor were cultural activities, politics, and social affairs and activities such as clubs and voluntary work. Factor loadings for all goal items were rather strong and well above 0.40, excluding social activities (i.e., clubs, voluntary work).

From the four factors, only the factor 1, the PA and sports, was found associated with walking (*β* = 0.167, *p* < 0.001). Thus, only the PA and sports goal factor was taken for further analysis.

### 3.2. Effects of Personal, Psychological and Environmental Features on Older Adults’ Walking for Transport

We examined how personal, psychological and environmental features predicted walking behavior in older adults. We tested separate OLS regression models for each of the five density measures. Table 4 presents the standardized beta coefficients of walking predicted by environmental variables after controlling for PA and personal variables. As shown in Table 4, income has a significant negative and education has significant positive associations with walking in all of the five models, thus retaining H1 only partially, as gender, marital status and perceived health have no significant direct associations with walking. PA and sports-related personal goals has significant positive effect on walking, retaining H2 partially. The psychological factors associate positively with older adults’ walking behavior (Table 3), but only those related to PA and sports as no other goal factors were found associating with walking for transport. As shown in Table 4, walkway density, intersection density, residential density, public transit stop density and density of sporting places have all significant positive associations with walking, thus retaining H3.

All of the five models resulted with alike outcomes. As shown by Table 4, income and education were found as the only personal characteristics having a direct effect on walking. In all five models, higher monthly income had a negative, but rather weak direct effect on walking, meaning that higher monthly income meant less walking to the everyday errand points. On contrary, higher education status had a positive yet also rather weak direct effect on walking, indicating that the higher the education status the more the respondent walked to access the EEP’s (Table 4).

**Figure 4:**
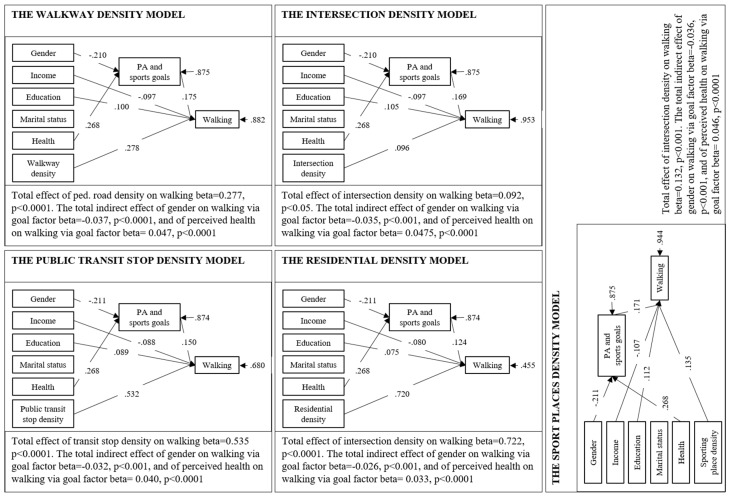
Significant paths with standardized Beta (*β*) for the five different theoretical models. The figure illustrates only the significant paths between different variables.

The studied psychological feature, the PA and sports goal factor, was found also having a direct effect on walking in each model, meaning that the higher score for PA and sports goals factor the person had the more they walked (Table 4). The environmental features, namely walkway density, intersection density, residential density, public transit stop density and the density of sporting places within the home range of each individual had all direct effect on walking and the direct effect of all the physical environmental features on walking was positive and quite large (Table 4 and Figure 4).

#### 3.2.1. Mediation Models

The indirect effects of personal as well as environmental variables on walking via PA and sport goal factor was examined using structural equation modeling. We tested five path models that all of them fitted the data perfectly (RMSEA = 0.00, CFI = 1.00, TLI = 1.00). Gender and perceived health were found having a significant indirect effect on walking through PA and sports goal factor in all of the five different models (Table 4 and Figure 4). Thus, the indirect effects of gender and perceived health are realized through PA and sports related personal goals retaining partially H4. The direct effect of gender on PA and sports goals varies very little model by model (from −0.210 to −0.211) and is negative, meaning that men, compared to women, had significantly less PA and sports related personal goal factor scores. The total indirect effect of gender on walking is significant and varies between −0.026 and −0.037 in the five different models (Figure 4), thus suggesting that the PA and sports goals mediate the effect on walking behavior between men and women.

The perceived health has a strong relationship with the PA and sports goals in all of the five models (0.268, *p* < 0.001) suggesting that older adults who perceive their overall health good report having PA and sports related personal goals. The total indirect effect of perceived health on walking is significant and varies between 0.033 and 0.0475 in the five different models (Figure 4), thus suggesting that personal goals related to PA and sports plays a mediating role in the relationship between perceived health and walking in older adults’.

As to the mediation analysis, we calculated the standardized estimation of direct, indirect and total effects of personal and environmental features on total walking with PA and sport goal factor as mediator. Figure 4 shows the results of significant direct and indirect paths. Walkway density, intersection density, residential density, public transit stop density and density of sporting places none have a significant effect on personal goals, thus rejecting the H5.

## 4. Discussion

The motivation for this study arose from the notions that research focusing on multiple-level influences on health behavior are still needed and that the health behavior of individuals is not bound to static neighborhood boundaries [11,40,71]. In addition, studies examining associations between older adults’ psychological factors and PA is lacking [14]. While ecological models have raised interest among researchers, productive frameworks that focus context-sensitively and simultaneously on both the individual and the physical environment are still infrequent [9,42].

In this study, we examined the associations between the personal, psychological and the environmental features with older adults active travel behavior with a spatial approach that takes into account the various different spatial scales of human mobility. We aimed to determine which individual and environmental features explain walking for transport among older adults. We found that several physical environment features had significant and positive direct effects on older adults walking. The psychological features examined did not have a mediating role in the relationship between the physical environment and walking in older adults’. Thus, the physical environment had an independent effect on active mobility regardless of individual demographic or psychological features. Walkway density, residential density, connectivity, namely the density of public transit stops and intersections, and the density of recreational sport places within respondents’ home ranges were significantly and positively associated with their walking for transport. Thus, the results suggest that the built environment plays a significant role in supporting walking of older adults, even for those not particularly interested in physical activities. From the physical environment features the residential and public transit stop density were found having largest direct effect to older adults walking. In the case of residential and public transit stop density their total effect to walking was clearly higher than the unexplained variance whereas the intersection and the sporting places density had rather small direct effect. However, all of these results support the previous findings about the independent effect of built environment to active travel of older adults [8,72]. Numerous studies have reported that well-connected, pedestrian friendly, and dense built environment influence positively mobility and physical activity of older adults [30]. However, in many of the previous studies, biased associations are possible because individuals who prefer an active lifestyle in general may seek to move around and live in areas of high walkability [73]. Our results add to the previous evidence by showing that the associations between built environment and walking behavior tend to exists even after controlling the motivational features behind people’s actions. Thus, the physical environment can play strong role for older adults’ walking behavior despite their personal interests and background.

Personal psychological features, namely the personal goals related to physical activity and sports, had a direct positive effect on walking, meaning that the higher the importance of physical activity and sports related goals were for the older adult the more they walked for transport. Based on the personal goals that the participants reported, we identified four goal factors. These factors included goals related to physical activity and sports, caring for others, managing on one’s own, and culture and social affairs. Only physical activity and sports was significantly associated with active mobility and the other three factors had no significant association. These results are in line with previous research where respondents who reported having personal goals related to exercise were found four times more likely to have high exercise activity than those who did not report exercise-related goals [22].

The physical activity and sports goals had also a mediating effect on the relationship between gender as well as perceived health and walking. Thus, our results further strengthen the notion that psychological factors are associated with physical activity in older adults [14] and that personal characteristics have indirect influences through personal goals to the outcome measures [28]. Previous studies have concluded that personal goals are potential for studying and representing the volitional process people use in choosing their everyday behaviors and are central to motivation [21]. Our results suggest that strong interest toward physical activity and sports can affect the active mobility behavior of a person.

We found income and education having a direct effect on walking for transport in older adults. Income was negatively associated with walking, meaning that higher the income the less the respondent walked for transport. However, this finding is not a major public health concern firstly because the direct effect was small and individuals with higher income have been shown replacing the lower transport walking behavior with other forms of physical activity [74,75]. In contrast, the lower socio-economic status has been linked to less recreational walking among older adults [76], whereas King and colleagues [77] found the neighborhood income not being associated with active transport. Education had a direct positive effect on walking, meaning that higher the education level the more the older adults walked for transport. Similar results have been found in studies among the general population, where higher education was found positively associated with frequency of transport-related walking where leisure-time physical activities explained the higher frequencies [74]. Higher levels of walking for transport in higher education groups could be explained here by the attitude towards, and adoption of, an active lifestyle similarly as by Cerin and colleagues [67]. However, these results warrant for careful considerations on the importance of health and physical activity education interventions among older adults [78]. In contrast to these findings, Cerin and colleagues [75] found respondents with higher education reporting lower levels of within-neighbourhood and overall transport walking in Hong Kong elders.

The individual home range modeling approach enabled us to study the characteristics of the environment within those exact geographical areas where the respondents live in and report moving around [56]. A majority of studies use plain administrative units or spherical buffers as geographical units of analysis when conducting research on built environment effects on health, and thus are susceptible to the uncertain geographic context problem [55,70]. Many studies still to date examine individual health behavior out of context, disconnected from the physical environment where the behavior actually takes place, or focus merely on personal perceptions of neighborhood characteristics [23,43,79]. The spatial dimensions and modeling techniques related to studies on the contextual effects have been shown to have a clear effect on the outcomes of studies but these should be more carefully examined in future research [39,40,59]. The future studies should take into careful consideration also the modifiable areal unit problem (MAUP), the uncertain geographic context problem (UGCoP) as well as the ways to measure the walkability of the environment in different contexts [59,70,80,81].

We acknowledge that our study has several limitations. The PPGIS methodology could be seen as causing limitations for the studied population group, as those with poor computer literacy or no access to internet could be excluded from the study. However, Finns are technologically well-oriented, and age does not play a significant role in their use of public e-services [82]. In addition, the suitability of the PPGIS method for older adults has been studied, and the results showed its applicability to both older adults and a wider audience, including people with low mapping experience and poor computer literacy [83]. Our walking for transport measure could be seen vulnerable to the bias of self-reporting. However, in a study by Crutzen and Göritz [84] no significant associations between social desirability and self-reported physical activity in web-based research was found. Measuring the destination density in more detail could have added value to the study, but due to data limitations this was not possible. Future research should focus in more detail to the destination density and their quality related to walking for transport [8,32,66]. The cross-sectional nature of this study can be also seen a one of the limitations.

## 5. Conclusions

We studied the associations between the personal, psychological and the environmental features and older adults walking. We examined the direct effects of the personal, psychological as well as environmental features on older adults walking as well as the indirect influences of environmental and personal characteristics through psychological features, namely individuals’ personal goals. Walkway density, residential density, connectivity, and the density of recreational sport places within respondents’ home ranges had an independent effect on older adults walking for transport regardless of individual demographic or psychological features. Residential and public transit stop density were found having largest direct effect to older adults walking. Thus, the walkable, well-connected and destination rich environment may encourage the walking behavior even of those who are not very interest in physical activities. Personal goals related to physical activity and sports had also a direct positive effect on walking. Additionally, we found an indirect effect of gender as well as of perceived health on walking which was realized through individuals’ physical activity and sports goals.

Future research should aim for longitudinal studies to more comprehensively examine causal relations and use other advanced data modeling among the studied variables, as suggested elsewhere [9]. According to our results and previous literature, we suggest that future studies on physical activity and health interventions should investigate simultaneously the personal and psychological as well as the physical environment features on human mobility with spatially bounded context-specific methods to be able to capture individuals’ true exposure to environmental influences [30,39,59,85].

## Figures and Tables

**Table 1:** The sociodemographic factors of respondents (*n* = 844).

Variable	Sample (%)	Statistics Finland (%) *
Gender		
Male	43	45
Female	57	55
Education ^a^		
Basic education	12	40
Upper secondary education	42	33
Lower university degree	15	11
Higher university degree	31	17
Age		
55–64	52	55
65–74	48	45
Housing		
Apartment	59	70
Detached/row house	41	30
Retired	60	59
Income (median) ^b^		
Ages 55–64	3501–4000	4001–4500
Ages 65–74	3001–3500	3001–3500

* The sample consists of Finnish people living in the capital area, aged 55–75, in 2015 (a and b exceptions). ᵃThe reference sample consists of Finnish people living in the capital area, aged 55+, in 2014. ᵇThe reference sample consists of all Finnish people aged 55–75 in 2014.

**Table 2:** Explanatory factor analysis.

Items	Factors			
	1. PA and Sports	2. Caring for Others	3. Manage on One’s Own	4. Culture and Social Affairs
Variance explained (%)	33	10	10	9
Everyday physical activities (e.g., walking, biking)	0.834			
Sports or dance hobby	0.682			
Maintaining health and functional capacity of the body	0.501		0.380	
Health and wellbeing of others		0.794		
Taking care of relatives		0.696		
Relationships		0.503		
Independent living, the preservation of an independent lifestyle			0.530	
Managing own financial issues and/or assets			0.505	
Maintaining memory capacities			0.486	
Cultural activities				0.627
Politics and social affairs			0.429	0.444
Social activities (i.e., clubs, voluntary work)				0.381

Note: Extraction Method: Principal Axis Factoring. Rotation Method: Promax with Kaiser Normalization.

**Table 3:** The descriptive statistics of the variables used in the analysis.

Variable	*n*	Mean	SD
Total walking ^a^	673	3.575	2.775
PA and Sports Goal Factor	693	14.685	3.094
Gender	693	0.431	0.495
Income	693	2.361	1.001
Education	693	2.631	1.045
Marital status	693	0.354	0.547
Perceived overall health	693	0.594	1.909
Walkway density ^a^	693	22.318	17.067
Intersection density ^a^	693	112.602	40.657
Public transit stop density	693	5.758	7.897
Residential density	693	1.203	3.548
Sporting places density	693	3.645	1.370

^a^ Measure was transferred using square root transformation because the data contained zero values.

**Table 4:** The standardized model results for direct and total indirect effects of predictors on walking via PA and sports goal factor.

Predictors	Physical Environmental Features				
	Walkway Density	Intersection Density	Residential Density	Public Transit Stop Density	Sporting Places Density
	*β*	*β*	*β*	*β*	*β*
**Gender ^a^**					
Direct effect ^b^	0.002	0.002	−0.002	0.000	0.000
Total indirect effect ^c^	−0.037 ***	−0.035 ***	−0.026 ***	−0.032 ***	−0.036 ***
**Income**					
Direct effect ^b^	−0.097 *	−0.097 *	−0.080 *	−0.088 *	−0.107 *
Total indirect effect ^c^	0.010	0.010	0.008	0.009	0.010
**Education**					
Direct effect ^b^	0.100 *	0.105 **	0.075 *	0.089 *	0.112 **
Total indirect effect ^c^	−0.010	−0.009	0.007	−0.008	−0.009
**Marital Status**					
Direct effect ^b^	−0.059	−0.040	−0.044	−0.042	−0.055
Total indirect effect ^c^	0.005	0.005	0.004	0.004	0.006
**Perceived Health**					
Direct effect ^b^	−0.055	−0.054	−0.043	−0.049	−0.055
Total indirect effect ^c^	0.047	0.045 ***	0.033 ***	0.040 ***	0.046 ***
**Environmental features ^d^**					
Direct effect ^b^	0.278 ***	0.092 *	0.720 ***	0.532 ***	0.135 **
Total indirect effect ^c^	−0.001	0.000	0.003	0.003	−0.003
Personal Goal F1 (PA, sports)	0.175 ***	0.169 ***	0.124 ***	0.150 ***	0.171 ***
R-Square	0.125 ***	0.125 ***	0.126 ***	0.126 ***	0.125 ***
RMSEA	0.000	0.000	0.000	0.000	0.000
GFI	1.000	1.000	1.000	1.000	1.000
TLI	1.000	1.000	1.000	1.000	1.000

* *p* < 0.05, ** *p* < 0.01, *** *p* < 0.001, ^a^ reference category = woman, ^b^ The direct effect of predictor on outcome after controlling for mediator (PA). ^c^ The effect of predictor on outcome via mediator. ^d^ the regression coefficients for environmental measures are divided by columns. *β* = standardized beta coefficient. DV: Total walking. RMSEA (Root Mean Square Error of Approximation), GFI (Goodness of Fit Indices), TLI (Tucker-Lewis index).
